# Plasma metabolomics and red blood cell fatty acid profiles in adolescent mental health

**DOI:** 10.1016/j.cpnec.2026.100347

**Published:** 2026-04-11

**Authors:** Aino-Kaisa Piironen, Alexey M. Afonin, Iman Zarei, Ville Koistinen, Marko Lehtonen, Venla Hämäläinen, Aleix Sala-Vila, Iolanda Lázaro, Jordi Julvez, Irene van Kamp, Katja M. Kanninen

**Affiliations:** a A.I. Virtanen Institute for Molecular Sciences, University of Eastern Finland, Yliopistonranta 1, P.O. Box 1627, FI-70211, Kuopio, Finland; b Institute of Public Health and Clinical Nutrition, School of Medicine, Faculty of Health Sciences, University of Eastern Finland, Yliopistonranta 3, FI-70211, Kuopio, Finland; c Institute of Biomedicine, School of Medicine, Faculty of Health Sciences, University of Eastern Finland, Kuopio, Finland; d Food Chemistry and Food Development Unit, Department of Biochemistry, University of Turku, Turku, Finland; e School of Pharmacy, Faculty of Health Sciences, University of Eastern Finland, Yliopistonranta 3, FI-70211, Kuopio, Finland; f Cardiovascular Risk and Nutrition, Hospital del Mar Medical Research Institute, Dr. Aiguader 88, 08003, Barcelona, Spain; g Centro de Investigación Biomédica en Red de la Fisiopatología de la Obesidad y Nutrición (CIBEROBN), Instituto de Salud Carlos III, Madrid, Spain; h Fatty Acid Research Institute, Sioux Falls, SD, USA; i Clinical and Epidemiological Neuroscience (NeuroÈpia), Institut d'Investigació Sanitària Pere Virgili (IISPV), Avinguda del Doctor Josep Laporte, 2, 43204, Reus, Spain; j Barcelona Institute for Global Health (ISGLOBAL), Barcelona, Spain; k Centre for Sustainability, Environment and Health, National Institute for Public Health and the Environment, Antonie van Leeuwenhoeklaan 9, 3721 MA, Bilthoven, the Netherlands

**Keywords:** Psychopathology, Biomarker, Global metabolomics, Liquid chromatography-mass spectrometry, Adolescents

## Abstract

Rising mental health challenges among adolescents are a global priority, yet tools to identify at-risk individuals remain limited. The development of objective tools, such as plasma biomarkers, could enhance the implementation of early preventive strategies for individuals at increased risk. We explored plasma metabolites associated with overall psychosocial difficulties in 197 adolescents aged 11-16 from the WALNUTS study. Psychosocial difficulties were assessed using self-reported Strengths and Difficulties Questionnaire (SDQ) scores. Plasma metabolomics data were generated by untargeted liquid chromatography high-resolution mass spectrometry using cross-sectional plasma samples. Linear regression modelling was performed to identify associations between plasma metabolites and the total SDQ score. Logistic regression and Precision-Recall curves were used to evaluate the classification performance of candidate metabolites in distinguishing the SDQ groups (low: 0–14; raised: 15–25). Additionally, pre-existing red blood cell fatty acid profile data were analysed to detect differences between the low and the raised SDQ score groups. Three metabolites: isoleucine, pregnenolone sulfate, and lysophosphatidylcholine 20:1, were significantly associated with the SDQ score, and are involved in energy metabolism, neuronal functions and phospholipid-related signalling. In addition, a trend towards lower proportions of red blood cell *n*-3 polyunsaturated fatty acids was observed in individuals with the raised SDQ score. This exploratory study observed associations between three plasma markers and the total SDQ score, reflecting psychosocial, behavioural, and emotional difficulties in adolescents.

## Highlights

• Three plasma metabolites associated with psychosocial difficulties in adolescents.
• Isoleucine was positively linked to psychosocial difficulties.
• Pregnenolone sulfate was negatively linked to psychosocial difficulties.
• Lower LPC 20:1/0:0 was linked to the highest SDQ scores.

## 1 Introduction

The mental well-being of children and adolescents is of great concern and has received increased global attention [1]. Mental health conditions are among the top 10 leading causes of disease burden worldwide [2]. Specifically, in young people aged 10-24 years, mental disorders affected 15.5% of the global population in 2021 [3]. Depressive and anxiety disorders were among the top seven leading causes of disability-adjusted life-years (DALYs) in 2019 [2].

The timing of mental health promotion and preventive strategies is crucial. Mental disorders, often with onset in adolescence, disturb psychosocial and emotional development, leading to a higher risk of morbidity, mortality and dysfunction in adulthood [4]. The assessment of mental health currently relies on subjective clinical interviews and validated questionnaires [5]. Thus, there is a lack of objective tools for the early identification of individuals with increased psychosocial difficulties and a high risk of mental disorders.

Despite the variety of symptoms of different mental disorders, common underlying biological processes have been reported in major psychiatric disorders such as depression, bipolar disorder and schizophrenia [6]. These disorders also show shared commonalities in genetic [7], psychosocial, and environmental risk factors such as childhood trauma and maltreatment, bullying victimisation, poverty, and substance abuse [4,8]. Furthermore, adolescent psychiatric disorders commonly show comorbidities due to, e.g. overlapping symptoms and heterogeneous manifestations of diseases [9]. Thus, a more general approach to mental health and illness is beneficial when aiming at the identification of adolescents at high risk or those with nonspecific symptoms.

The Strengths and Difficulties Questionnaire (SDQ) is a screening tool to evaluate emotional, behavioural and psychosocial difficulties and prosocial behaviour in children and adolescents aged 4-17 [10,11]. Children with high SDQ scores have been shown to have an increased probability of clinical mental disorders [12]. Recognised as a validated psychometric instrument widely employed in clinics, the SDQ score serves as a quantitative indicator for assessing psychosocial difficulties in children and adolescents.

In addition to psychometric tools like the SDQ, recent advancements in biological research have revealed potential biochemical markers of mental health, complementing traditional screening methods. Over the past decade, interest in metabolomics has grown within psychiatric research [13]. However, previous studies have mostly focused on disease-specific targeted approaches [[14], [15], [16]] while relatively few studies on adolescent mental health have employed untargeted metabolomics, which has the advantage of unbiased discovery of novel biomarkers and pathways in a hypothesis-generating manner [[17], [18], [19]]. Prior plasma metabolomics studies have revealed alterations in lipid metabolism, especially reduced levels of polyunsaturated fatty acids (PUFA), in adolescents with depression [14,19]. Recently, shared differences between all major psychiatric disorders and healthy adolescents were identified in fatty acid, steroid-hormone, purine, nicotinate and amino acid metabolism [18]. Similarly, dysregulation of lipid, amino acid and energy metabolism was found in the urinary metabolome of children and adolescents with depression [20].

This study aimed to identify plasma metabolites associated with psychosocial difficulties in adolescents assessed by the total SDQ score. Although lipid and amino acid metabolism have been increasingly recognised as biomarkers of mental health [18,19], the underlying biological mechanisms and early pathophysiological indicators remain poorly understood. Untargeted plasma metabolomics offers a comprehensive approach to discover novel biomarker candidates by exploring the plasma metabolome without prior knowledge of specific target metabolites [17]. We hypothesised that this holistic method could reveal biomarker candidates for early psychopathology and provide insights into the complex biological networks underlying the physiology of mental health.

## 2 Materials and methods

The methods and study population are described in detail in Appendix A (Supplementary Methods).

### 2.1 Plasma samples and psychosocial status

The cross-sectional peripheral blood plasma samples were obtained and analysed from 197 adolescents aged 11-16 years participating in the WALNUTS regional Spanish study (Table 1, Table A.1) [21,22]. The samples were collected in 2016-2018, close to the moment participants filled out the Strengths and Difficulties Questionnaire (SDQ). Samples were drawn by a nurse using K2EDTA plus tubes, rested for 1 h and then centrifuged at 2500×*g* for 20 min at 20 °C, refrigerated at 4 °C, and frozen to −80 °C within 4 h after extraction [22]. The specifics of the cohort formation were described in previous publications [21,22]. The self-reported SDQ score was used to assess the psychosocial status. Based on the total SDQ score, the plasma samples were categorised into low (SDQ = 0-14) and raised (SDQ = 15-25) groups [23]. Additional sociodemographic and lifestyle factors of the study population are presented in Table A.2. The studies were reviewed and approved by the CEIC Parc Salut Mar Clinical Research Ethics Committee (approval nos. 2015/6026, WALNUTs; 2020/9688, Equal-Life). Written informed consent to participate in the original WALNUTs study was provided by the participant's legal guardian/next of kin. No additional consent was needed for this study.

**Table 1:** Sample characteristics by the SDQ score groups.

Group	Low SDQ score (0-14)		Raised SDQ score (15-25)	
**Sex**	**Female**	**Male**	**Female**	**Male**
**Sample size** n (%)	146 (74.1)		51 (25.9)	
	63 (43.2)	83 (56.8)	22 (43.1)	29 (56.9)
**SDQ scores** mean ± s.d.	7.83 ± 3.88		17.39 ± 2.25	
	7.41 ± 3.66	8.14 ± 4.03	18.00 ± 2.43	16.93 ± 2.02
**Age** (years) mean ± s.d.	13.88 ± 0.86		14.15 ± 1.09	
	13.93 ± 0.83	13.84 ± 0.88	14.38 ± 1.13	13.97 ± 1.04
**BMI** (kg/m^2^) mean ± s.d.	20.36 ± 3.40		20.81 ± 3.30	
	20.56 ± 3.50	20.21 ± 3.32	21.15 ± 2.59	20.56 ± 3.77
**Psychiatric/neuropsychiatric/neurological** **diagnoses** n (%)	5 (3.4)		5 (9.8)	
	<5	<5	<5	<5
**Fasting time** (hours)**,** mean ± s.d.	3.3 ± 3.03		3.8 ± 3.73	
	3.1 ± 2.63	3.5 ± 3.31	4.4 ± 3.9	3.4 ± 3.63
**Medication use (any)** n (%)	13 (8.9)		<5 (<9.8)	
	6 (9.5)	7 (8.4)	<5	<5
**Alcohol usage** (yes), n (%)	51 (34.9)		22 (43.1)	
	26 (41.3)	25 (30.1)	10 (45.5)	12 (41.4)
**Smoking∗ (yes),** n (%)	13 (8.9)		8 (15.7)	
	8 (12.7)	5 (6.0)	3 (13.6)	5 (17.2)

n = number of samples (% of each group); s.d. = standard deviation, SDQ = the Strengths and Difficulties Questionnaire, BMI = body mass index (kg/m^2^), ∗smoking normal cigarettes.

### 2.2 Fatty acids analysis

Fatty acid profiles in red blood cells (RBC) were determined by gas chromatography using an Agilent HP 7890 Gas Chromatograph equipped with a 30 m × 0.25 μm x 0.25 mm SupraWAX-280 capillary column (Teknokroma, Barcelona, Spain), an autosampler, and a flame ionisation detector [22]. The amount of each fatty acid was expressed as a percentage of total fatty acids in the sample. The omega-3 index was calculated as the sum of eicosapentaenoic (EPA) and docosahexaenoic (DHA) acids in RBC membranes and expressed as a percentage of total fatty acids. Similarly, the percentages of fatty acid groups were calculated as a sum of saturated (SFA), monounsaturated (MUFA), *n*-3 polyunsaturated (*n*-3 PUFA), and *n*-6 polyunsaturated fatty acids (*n*-6 PUFA). Data on fatty acid profiles were available from all 197 individuals in this subcohort. The detailed information is described in Ref. [22,24].

### 2.3 Liquid chromatography - mass spectrometry analyses

Preprocessing of the plasma samples (see Appendix A) followed by untargeted metabolite profiling was conducted at the Biocenter Kuopio LC-MS metabolomics facility (University of Eastern Finland, Finland). The analysis was conducted using an ultra-high-performance liquid chromatography (UHPLC) system (Vanquish Flex, Thermo Scientific, Bremen, Germany). The liquid chromatography system was integrated online with a high-resolution mass spectrometer (HRMS, Q Exactive Focus, Thermo Scientific). All samples were examined for metabolomics analysis using two distinct chromatographic techniques: reversed phase (RP) and hydrophilic interaction chromatography (HILIC), and both electrospray ionisation polarities (ESI+/−). Data-dependent product ion spectra for the identification of molecular features were obtained from pooled quality control (QC) samples at the start and end of the analysis for each mode. Additionally, QC samples were included in the analysis at the beginning of the worklist and after every 12 samples. The configuration and specifications of the LC-MS instrument have been previously documented [25].

### 2.4 Data pre-processing

Raw spectral data of each mode (HILIC/RP, ESI+/−) were preprocessed in Compound Discoverer software (v.3.3, Thermo Scientific, CA, USA) using the template of Untargeted Metabolomics with Statistics Detect Unknowns with ID using Online Databases and mzLogic [26]. Using the original raw data obtained in untargeted LC-HRMS, a semi-targeted approach was carried out to detect metabolites (n = 152, Table B.1) previously documented in biomarker studies on mental health in young people [[18], [19], [20],[27], [28], [29]]. Data pre-processing for the semi-targeted approach was performed similarly for HILIC and RP positive data as described above but complemented with an additional node to accomplish a search against the list of known metabolites, including metabolite name, formula and mass (Table B.1).

Data from untargeted and semi-targeted mass list analyses were further processed in R (R Core Team, v.4.3.1). Principal component analysis was performed to assess the general quality of data (Figure A.1). Detection threshold filtering was performed separately for low and raised SDQ samples to select metabolite features with sufficient intensity levels across all samples (Figure A.2). Using the filtered data, quantile normalisation and log2 transformation were performed. The ggplot2 (v.3.5.1) was used for data visualisation.

### 2.5 Statistical analysis

Data processing and statistical analyses were performed using R (v.4.3.2). The *limma* [30] package (v.3.56.2) was used for linear regression modelling using combined data from all modes, but separately for untargeted and semi-targeted mass list metabolomics. The total SDQ score was included as a continuous variable, and age, sex, BMI, and fasting time were covariates in regression models. Missing values for fasting time (two missing) were imputed using the median, and a sex-specific median was used for BMI (two missing values). As a second model to detect possible non-linear associations between molecular features and the total SDQ score, splines were included in *limma* modelling using the same covariates. Sensitivity analyses were additionally adjusted for medication use, alcohol consumption, and smoking in separate models, including participants with available data (n = 193, 174, and 171, respectively). RBC fatty acid profiles were analysed using raised vs low SDQ groups in Welch's *t*-test and linear regression (*limma* with makeContrasts-function) with age, sex and BMI as covariates. Single fatty acids with skewed distributions were expressed in log2-transformed values. Non-transformed values were used for percentages of the fatty acid groups and the omega-3 index. For all analyses, the false discovery rate (FDR), reported as *q*-values, was computed using the Benjamini–Hochberg method [31], and statistical significance was considered for *q*-values less than 0.05. The abovementioned metabolomics and fatty acid analyses included data of all 197 participants.

Further examination of top candidates (*q* < 0.05, MS/MS data, and level 2 annotations as described in section *2.6 Feature annotations*) was performed using multiple linear and binary logistic regression with Cook's distance to identify potential outliers that strongly influenced the regression parameters. Precision-Recall curves (PR curve) with 5-fold stratified cross-validation using the *PRROC* package [32,33] (v.1.4) were generated to assess the classification performance of the raised SDQ group in binary logistic regression models.

### 2.6 Feature annotations

Top metabolite features were selected for annotation based on statistical analysis, focusing on features with MS/MS data available. Feature annotations were performed as described in previous publications [34,35]. Briefly, MS-DIAL [36] was utilised in the first semi-automated step of metabolite identification to compare the experimental characteristics, including exact *m/z* (mass-to-charge ratio*),* retention time (RT) and MS/MS spectra, with those in databases (METLIN, MassBank of North America (MoNA), Human Metabolome Database (HMDB), LIPID MAPS), and the in-house spectral library [34]. After semi-automated annotations using MS-DIAL, additional searches were performed for remaining unknown metabolites using online databases such as LIPID MAPS [37] and MS-FINDER [36]. Annotated metabolites were classified into four levels according to the scheme of the Metabolomics Standards Initiative (MSI) [38]. In the untargeted LC–MS workflow, all detected features were retained during preprocessing and statistical modelling. Feature grouping and redundancy reduction were performed at the annotation stage as described in Appendix A.

## 3 Results

### 3.1 Association of metabolites with the total SDQ score in the untargeted analysis

**Fig. 1:**
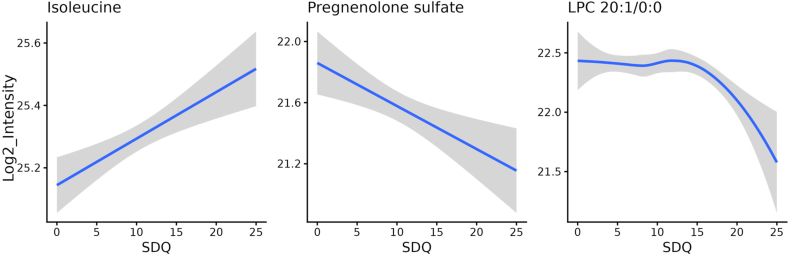
**Metabolites associated with the SDQ total score.** The blue line represents the trend line, generated by linear modelling or LOESS (Locally Weighted Scatterplot Smoothing), and the grey area indicates the 95% confidence interval. Isoleucine was identified as significant in semi-targeted mass list analysis, and other metabolites in untargeted analysis.

Among 1992 metabolite features included in the linear modelling, we identified 6 features significantly (*q* < 0.05) associated with the total SDQ score, of which two candidate metabolite biomarkers were successfully annotated (Table 2). The annotated significant metabolites included pregnenolone sulfate with a negative linear relationship with the SDQ score (*q* = 0.029, linear effect size = −0.038), and a long-chain lysophosphatidylcholine (LPC), LPC 20:1/0:0, with a nonlinear association (*q* = 0.032, Fig. 1, Figure C.1). Of all the significant features, two features had a linear association, and four features had a non-linear association with the SDQ (Table C.1).

**Table 2:** **Metabolites significantly associated with the total SDQ score.** Linear effect size is the log2-fold change in intensity resulting from a unit (1 score) change in the total SDQ score. Data of all participants (n = 197) were included in the analyses.

HMDB ID/LIPID MAPS ID	Metabolite name	MSI ID level	Linear effect size	Linear *p*-value	Linear *q*-value	Non-linear *p*-value	Non-linear *q*-value	Mode
Untargeted analysis								

Metabolites with a linear association with the SDQ score								
HMDB0000774, HMDB0060382/LMST05020014	Pregnenolone sulfate	2	−0.038	2.8E-5	**0.029**	3.5E-4	0.079	RP-
Metabolites with a non-linear association with the SDQ score								
HMDB0010391	LPC 20:1/0:0	2	**-**	2.6E-3	0.085	9.5E-5	**0.032**	RP+
Semi-targeted mass list analysis								

Metabolites with a linear association with the SDQ score								
HMDB0000172	Isoleucine	2	0.015	2.0E-4	**0.034**	1.3E-3	0.134	RP+

HMDB = the Human Metabolome Database, MSI = Metabolomics Standards Initiative, SDQ = the Strengths and Difficulties Questionnaire, LPC = Lysophosphatidylcholine. RP- = reversed phase chromatography, negative ionisation, RP+ = reversed phase chromatography, positive ionisation.

### 3.2 Semi-targeted mass list analysis

As a complementary approach to utilise the untargeted metabolomics data, we performed semi-targeted mass list analysis with 152 predefined metabolites of interest (Table B.1). Linear and non-linear modelling identified 13 associations with a nominal *p*-value <0.05 (Table C.2). Of those, only isoleucine in RP positive data remained significant (*q* = 0.034, linear effect size = 0.015) after multiple testing corrections (Fig. 1, Table 2, Table C.2).

### 3.3 Analysis of top candidate metabolites

**Fig. 2:**
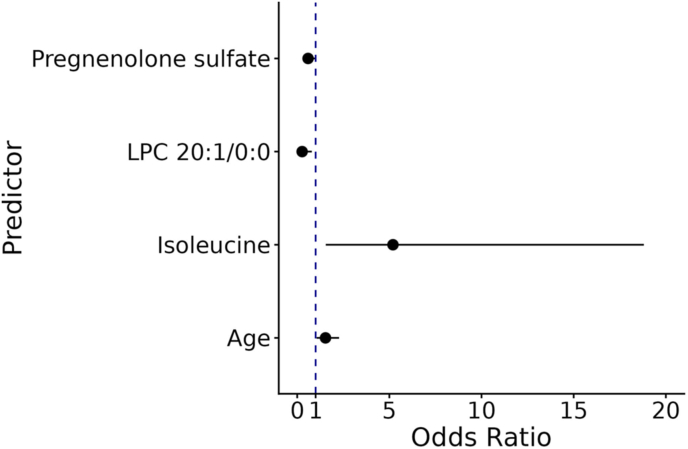
**The Odds Ratios of the candidate metabolites and age.** The dot represents the odds ratio, and the horizontal line indicates the 95% confidence interval. The odds ratios represent the change in the odds of the outcome (the raised SDQ group) for a one-unit increase in log2-intensity of a metabolite, or a one-year increase in age.

The criteria for the top candidate metabolites included statistical significance (*q* < 0.05), MSI level 2 annotation, and MS/MS data. Further examination of the top candidate metabolites, namely isoleucine, LPC 20:1/0:0 and pregnenolone sulfate, revealed that the linear regression model consisting of isoleucine, pregnenolone sulfate, LPC 20:1/0:0 (as a linear predictor), and age was significantly associated with the total SDQ score (R^2^ = 0.19, *p*-value = 4.2E-08) (Table C.3). Furthermore, six logistic regression models without one expected outlier (Figure C.2) were generated to distinguish the SDQ groups (raised vs low) (Table C.4). The odds ratios of the model including the three candidate metabolites and age are presented in Fig. 2. The area under the PR curves (AUPRC) showed similar classification performance across the models (AUPRC 0.66-0.71, baseline 0.26), suggesting that the key metabolites with age could reasonably distinguish the raised SDQ group (Figure C.3).

### 3.4 Sensitivity analyses

Additional regression models using both untargeted and semi-targeted mass list data confirmed significant (*q* < 0.05) associations of isoleucine with the SDQ score after adjusting for medication use, alcohol consumption, and smoking. LCP 20:1/0:0 was significant in sensitivity models adjusted for medication use and smoking, and pregnenolone sulfate remained significant after adjusting for medication use, but not in the other models. None of the key metabolites significantly correlated with fasting time. The results are presented in Table C.5.

### 3.5 Fatty acid measurements

**Fig. 3:**
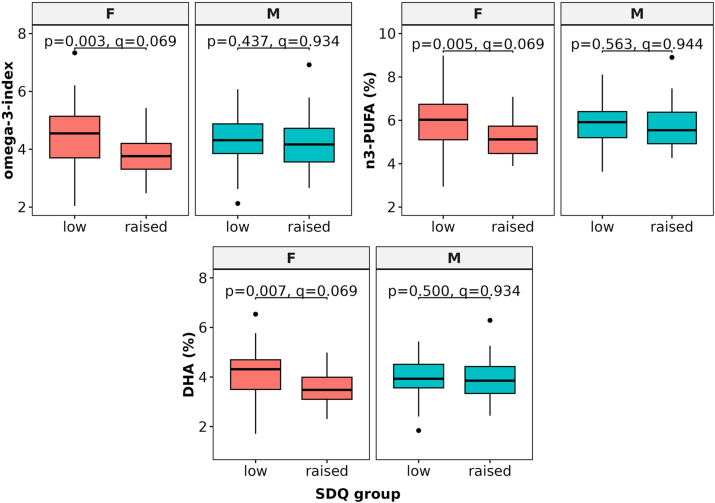
**Differences in the omega-3-index and proportions of *n*-3 PUFAs and docosahexaenoic acid (DHA) between the SDQ groups.** Statistical significance in the Welch's *t*-test. PUFA = polyunsaturated fatty acids, SDQ = the Strengths and Difficulties Questionnaire.

We used pre-existing data on RBC fatty acid profiles, which were determined in a previous study of the WALNUTS cohort [22]. Nominally significant differences in fatty acid profiles between the raised and low SDQ score groups were found in omega-3 fatty acids, i.e., *n*-3 FAs (Fig. 3, Table C.6A-C). Among all samples (n = 197), the proportions of EPA, DHA, *n*-3 PUFA, and omega-3 index were slightly lower in the raised SDQ group in both analyses (Table C.6A). Similarly in girls (n = 85), nominally significant differences were seen in DHA, *n*-3 PUFA and omega-3 index (Table C.6B), whereas in boys (n = 112), docosapentaenoic acid (DPA) was slightly lower in the raised SDQ group (Table C.6C). Age, BMI, fasting time and medication use showed only weak correlations with fatty acids (Figure C.4).

## 4 Discussion

Plasma metabolomics has been increasingly applied in biomedical research of psychiatric diseases. To our knowledge, this is the first study to investigate plasma metabolites associated with psychosocial difficulties in adolescents as indexed by the total SDQ score. Using untargeted liquid chromatography-mass spectrometry, we identified three candidate metabolites (MSI level 2) significantly associated with the total SDQ score in adolescents. Precisely, isoleucine was increased, and pregnenolone sulfate and LPC 20:1/0:0 were decreased in adolescents with higher SDQ scores, indicating increased psychosocial, behavioural, and emotional difficulties in daily life. Additionally, a trend towards lower RBC *n*-3 PUFAs was observed in the raised SDQ group.

One of the top candidates observed in this study was isoleucine. Previous studies have reported on the role of branched-chain amino acids (BCAA) in mental disorders [39]. Proteinogenic BCAAs, including valine, leucine and isoleucine, are essential amino acids involved, e.g. in protein synthesis and energy metabolism [40]. We identified a positive association of isoleucine with the SDQ score. Similarly, higher plasma levels of isoleucine were positively associated with depression and atypical, energy-related depressive phenotype in adults [41,42]. Furthermore, the positive, although not significant, trend between isoleucine and depressive symptoms was seen in young adults [28]. BCAAs and aromatic amino acids (phenylalanine, tyrosine, tryptophan) are large neural amino acids with shared transporters, such as LAT-1, across the blood-brain barrier. Considering this competitive transport, increased plasma isoleucine might contribute to the neurotransmitter synthesis of serotonin, dopamine, and norepinephrine by influencing the availability of precursors such as tryptophan. [40]. While the mechanisms of isoleucine in psychopathology remain unclear, our findings suggest an association of BCAA-driven energy metabolism with psychosocial difficulties in adolescents.

The second candidate associated with the total SDQ score was LPC 20:1/0:0. LPCs are the most abundant lysoglycerophospholipids in human blood and perform a variety of roles in cell signalling and transport. They are essential for brain growth, likely for fatty acid delivery during brain development and growth. [43]. Interestingly, plasma long-chain LPCs can cross the blood-brain barrier via the major facilitator superfamily domain-containing protein 2A (Mfsd2a) transporter [44], and they were suggested as a preferred physiological carrier of DHA to the brain [44,45]. We identified a reduction in LPC 20:1/0:0 and marginally lower DHA, suggesting potential dysfunction in *n*-3 fatty acid transport in adolescents with psychosocial difficulties. However, this hypothesis necessitates further research. In a previous study relating to adolescent mental health, certain plasma long-chain LPCs were higher in children who had psychotic experiences (PE) six years later compared to controls without PEs [27]. While current evidence suggests alterations in LPC metabolism in youth mental health, additional research is necessary to elucidate whether and how dysregulation of specific long-chain LPCs may influence biological processes, potentially contributing to the psychosocial difficulties and development of psychopathology.

Additionally, this study observed a negative association of pregnenolone sulfate with psychosocial difficulties in adolescents. Pregnenolone sulfate is a neurosteroid derived from pregnenolone, which is the precursor of the steroid synthesis pathway, modulating a variety of ion channels, transporters, and enzymes [46]. For example, it enhances neuronal activity by inhibiting GABAergic and stimulating glutamatergic neurotransmission [47]. Due to its broad influence, pregnenolone sulfate significantly impacts brain functions, including cognitive enhancement, memory, neuronal development, and antistress and antidepressant effects [46,47]. In a study using a schizophrenia-like mouse model, peripheral injection of pregnenolone sulfate normalised the schizophrenia-like behaviour and preserved cognitive functions [48]. A decrease in pregnenolone sulfate in adolescents with higher SDQ scores could indicate an increased risk of psycho-cognitive challenges, making it a candidate target for further investigation.

Finally, we utilised data on RBC fatty acid profiles, reflecting the intake of dietary fats [49]. RBC membrane fatty acid profiles were previously determined and associated with attention scores by Pinar-Marti et al. [22], showing a positive association between DHA and attention performance. We observed a trend towards lower proportions of *n*-3 PUFAs, consisting of DHA and EPA, and the omega-3 index in adolescents with higher SDQ scores. However, these results should be interpreted with caution due to the insufficient statistical support. In previous studies, *n*-3 FA deficiency has been associated with the risk of neurodevelopmental disorders, behavioural and learning problems [50], and reported in depression [51]. Furthermore, *n*-3 FAs and major depressive disorder were genetically linked via *FADS* genes, demonstrating the role of the *n*-3 FA synthesis, and a potential indication of targeted prevention with *n*-3 supplementation [52].

Our study has several strengths, including the use of global metabolomics, which allows the identification of novel metabolite candidates, and the inclusion of RBC fatty acid profiles, which provide a longer-term indicator of dietary lipid intake. However, potential confounding factors such as dietary habits, physical activity, socioeconomic status, and genetic influences were not comprehensively accounted for, which may influence the observed associations. Furthermore, the associations of LPC 20:1/0:0 and pregnenolone sulfate with the SDQ score were confounded by alcohol consumption (for both) and smoking (for the latter). However, these lifestyle factors may also function as behavioural coping responses to increased psychosocial stress. Thus, the complex interactions among the observed lifestyle factors, plasma metabolites, and mental health outcomes necessitate further investigation in future studies.

As additional limitations, only a minority of participants in the WALNUTS study followed the required minimum of 8 h of fasting. Thus, we included the reported fasting time in regression modelling to minimise bias due to non-fasting samples. Furthermore, the fasting/feeding state does not affect the fatty acid composition of RBC fatty acids. Although the candidate metabolites were not significantly correlated with fasting time, the impact of meals or snacks before blood sampling cannot be reliably ruled out. For example, plasma isoleucine level rapidly increases after ingestion of protein-rich foods and can remain elevated for several hours [53]. Additionally, the candidate metabolites were not confirmed with authentic chemical standards and therefore, reported annotations correspond to MSI Level 2 (putatively annotated compounds). Future targeted validation using authentic standards is required to confirm these identities at MSI Level 1.

Altogether, our results represent a set of metabolites previously linked to mental health or brain functions. Alterations observed in plasma metabolites suggest possible biological changes related to lipid-mediated signalling and energy metabolism. While our findings provide insights into potential metabolic pathways involved in adolescent mental health, the cross-sectional design limits causal inference. Longitudinal studies incorporating repeated metabolomic assessments and intervention trials are necessary to confirm these findings and elucidate potential metabolic mechanisms underlying adolescent psychopathology.

In conclusion, our findings display biological pathways potentially implicated in youth mental health. By employing a global metabolomics approach, this exploratory study contributes to the growing body of evidence and provides hypotheses for future research on adolescent psychopathology. Validation of the identified candidate metabolites in adolescent mental health will require longitudinal studies that integrate additional omics layers and relevant environmental risk factors.

## CRediT authorship contribution statement

**Aino-Kaisa Piironen:** Writing – review & editing, Writing – original draft, Visualization, Formal analysis, Data curation, Conceptualization. **Alexey M. Afonin:** Writing – review & editing, Validation, Methodology. **Iman Zarei:** Writing – review & editing, Data curation. **Ville Koistinen:** Writing – review & editing, Data curation. **Marko Lehtonen:** Writing – review & editing, Resources, Methodology. **Venla Hämäläinen:** Writing – review & editing. **Aleix Sala-Vila:** Writing – review & editing, Investigation. **Iolanda Lázaro:** Writing – review & editing, Investigation. **Jordi Julvez:** Writing – review & editing, Resources, Investigation. **Irene van Kamp:** Writing – review & editing, Supervision, Project administration, Funding acquisition. **Katja M. Kanninen:** Writing – review & editing, Supervision, Project administration, Funding acquisition, Conceptualization.

## Declaration of generative AI and AI-assisted technologies in the manuscript preparation process

During the preparation of this work, the author(s) used ChatGPT in order to improve the readability and language of the single parts of the manuscript. After using this tool/service, the author(s) reviewed and edited the content as needed and take(s) full responsibility for the content of the published article.

## Funding

This project has received funding from the European Union's Horizon 2020 research and innovation programme under grant agreement No 874724. Equal-Life is part of the European Human Exposome Network. Moreover, the current work was supported by the European Union's Horizon 2020 research and innovation programme, grant number 874739 for the LongITools project. Aino-Kaisa Piironen has received personal working grants from the Finnish Cultural Foundation and the Yrjö Jahnsson Foundation.

## Declaration of competing interest

The authors declare the following financial interests/personal relationships which may be considered as potential competing interests:

Aino-Kaisa Piironen reports financial support was provided by Finnish Cultural Foundation. Aino-Kaisa Piironen reports financial support was provided by Yrjö Jahnsson Foundation. If there are other authors, they declare that they have no known competing financial interests or personal relationships that could have appeared to influence the work reported in this paper.

## Data Availability

The data that support the findings of this study are available from the corresponding author upon reasonable request. The data analysed in this study are subject to the following licenses/restrictions: The WALNUTS data is not publicly available due to the restrictions of informed consent. The data contains personal information of children/adolescents, and according to the ethical approval, they should be kept confidential. To ensure the protection of privacy and compliance with national data protection legislation, a data use/transfer agreement is needed, the content and specific clauses of which will depend on the nature of the requested data.
