# Supplementary material for: Plasma metabolomics and red blood cell fatty acid profiles in adolescent mental health
Source: Compr Psychoneuroendocrinol. 2026 Apr 11;26:100347. doi: 10.1016/j.cpnec.2026.100347 (PMC13092191; doi:10.1016/j.cpnec.2026.100347)
Supplement: Multimedia component 1 [file mmc1.docx]

**Supplementary Material A - Methods**

**Plasma metabolomics and red blood cell fatty acid profiles in adolescent mental health**

Aino-Kaisa Piironen, Alexey M. Afonin, Iman Zarei, Ville Koistinen, Marko Lehtonen, Venla Hämäläinen, Aleix Sala-Vila, Iolanda Lázaro, Jordi Julvez, Irene van Kamp, and Katja M. Kanninen

**Methods in detail**

**Table A.1**

**Table A.2**

**Figure A.1**

**Figure A.2**

**Methods in detail**

**Plasma samples and psychosocial status**

**Table A.1. Sample characteristics by three SDQ score groups.** The raised SDQ group was formed by combining the borderline and the high SDQ groups due to the low sample sizes.

| **Group** | **Low SDQ score (0-14)** | | **Borderline SDQ score (15-17)** | | **High SDQ score (18-25)** | | |
| --- | --- | --- | --- | --- | --- | --- | --- |
| **Sex** | **Female** | **Male** | **Female** | **Male** | **Female** | **Male** | |
| **Sample size**  (n (%)) | 146 (74.1) | | 31 (15.7) | | 20 (10.2) | | |
|  | 63 (43.2) | 83 (56.8) | 10 (32.3) | 21 (67.7) | 12 (60.0) | | 8 (40.0) |
| **SDQ scores**  (mean ± s.d.) | 7.83 ± 3.88 | | 15.94 ± 0.81 | | 19.65 ± 1.84 | | |
|  | 7.41 ± 3.66 | 8.14 ± 4.03 | 16.00 ± 0.82 | 15.90 ± 0.83 | 19.67 ± 2.02 | | 19.63 ± 1.69 |
| **Age** (years)  (mean ± s.d.) | 13.88 ± 0.86 | | 14.11 ± 1.08 | | 14.20 ± 1.13 | | |
|  | 13.93 ± 0.83 | 13.84 ± 0.88 | 14.28 ± 1.09 | 14.03 ± 1.10 | 14.46 ± 1.21 | | 13.81 ± 0.94 |
| **BMI** (kg/m^2^)  (mean ± s.d.) | 20.36 ± 3.40 | | 20.38 ± 3.55 | | 21.49 ± 2.83 | | |
|  | 20.56 ± 3.50 | 20.21 ± 3.32 | 20.23 ± 2.71 | 20.45 ± 3.94 | 21.92 ± 2.33 | | 20.84 ± 3.51 |

n = number of samples (% of each group); s.d. = standard deviation, SDQ = the Strength and Difficulties Questionnaire, BMI = body mass index (kg/m^2^)

The average fasting time was 3.5 ± 3.23 hours, and it was similar between the low and raised SDQ groups (3.3 ± 3.03 hours and 3.8 ± 3.73 hours, respectively). Overall, 19 participants fasted for over 8 hours.

Self-reported neuropsychiatric or neurological diagnoses included attention deficit hyperactivity disorder (ADHD) (three with low SDQ and one with borderline/raised SDQ), autism spectrum disorder (one with high SDQ), depression (one with borderline/raised SDQ), anxiety (one with borderline/raised SDQ), conduct disorder (one with high SDQ), and "other" psychiatric/neuropsychiatric disease (two with low SDQ) participants. Among these participants, three adolescents with ADHD and one with epilepsy reported medication use.

Pubertal status was determined based on self-reported variables: period for girls and voice changes for boys (Table A.2). Additionally, self-reported height growth and body hair development were available and reported at a Likert-scale: 1) It hasn't started to grow yet, 2) It has just started to grow, 3) It is growing a lot, 4) It looks like it has finished growing, 5) I don't know. The number and percentages of combined answers 3-4 by sex are reported in Table A.2.

**Table A.2 Confounding variables for low and raised SDQ groups.**

| **Group** | **Low SDQ score (0–14), n=146** | | **Raised SDQ score (15–25), n=51** | |
| --- | --- | --- | --- | --- |
| **Sex** (n) | **Female** (63) | **Male** (83) | **Female** (22) | **Male** (29) |
| puberty: period/voice change (yes),  n (%) | 51 (81.0) | 60 (72.3) | 19 (86.4) | 17 (58.6) |
| puberty: height growth (yes), n (%) | 32 (50.8) | 40 (48.2) | 12 (54.5) | 12 (41.4) |
| puberty: body hair (yes), n (%) | 35 (55.6) | 27 (32.5) | 15 (68.2) | 9 (31.0) |
| electronic_cigarette (yes), n (%) | 5 (3.4) | | < 5 (< 9.8) | |
|  | < 5 | < 5 | 0 (0) | < 5 |
| smoke_weed (yes), n (%) | < 5 (< 3.4) | | < 5 (< 9.8) | |
|  | < 5 | < 5 | < 5 | < 5 |
| other_drugs (yes), n (%) | < 5 (< 3.4) | | 0 (0) | |
|  | 0 (0) | < 5 | 0 (0) | 0 (0) |
| diet_following (yes), n (%) | 11 (7.5) | | < 5 (< 9.8) | |
|  | 6 (9.5) | 5 (6.0) | 0 (0) | < 5 |
| week_sleeping_time (hours) mean ± s.d | 8.0 ± 0.80 | | 7.6 ± 1.16 | |
|  | 8.0 ± 0.84 | 8.1 ± 0.78 | 7.6 ± 1.07 | 7.7 ± 1.23 |
| weekend_sleeping_time (hours) mean ± s.d | 9.4 ± 1.24 | | 9.3 ± 1.90 | |
|  | 9.4 ± 1.21 | 9.3 ± 1.27 | 9.8 ± 1.67 | 8.9 ± 2.00 |
| high-intensity physical activity: 1-2 times a week, n (%) | 60 (41.1) | | 25 (49.0) | |
|  | 32 (50.8) | 28 (33.7) | 15 (68.2) | 10 (34.5) |
| high-intensity physical activity: 3+ times a week, n (%) | 86 (58.9) | | 26 (51.0) | |
|  | 31 (49.2) | 55 (66.3) | 7 (31.8) | 19 (65.5) |
| mother_civilstatus, Married or stable couple, n (%) | 119 (81.5) | | 37 (72.5) | |
|  | 56 (88.9) | 63 (75.9) | 15 (68.2) | 22 (75.9) |
| mother_civilstatus, Separated/divorced, n (%) | 19 (13.0) | | 10 (19.6) | |
|  | 7 (11.1) | 12 (14.5) | < 5 | 6 (20.7) |
| father_civilstatus, Married or stable couple, n (%) | 120 (82.2) | | 36 (70.6) | |
|  | 56 (88.9) | 64 (77.1) | 15 (68.2) | 21 (72.4) |
| father_civilstatus, Separated/divorced, n (%) | 15 (10.3) | | 9 (17.6) | |
|  | 5 (7.9) | 10 (12.0) | < 5 | 5 (17.2) |
| mother_studies, university level n (%) | 95 (65.1) | | 31 (60.8) | |
|  | 42 (66.7) | 53 (63.9) | 11 (50.0) | 20 (69.0) |
| father_studies, university level n (%) | 88 (60.3) | | 23 (45.1) | |
|  | 39 (61.9) | 49 (59.0) | 7 (31.8) | 16 (55.2) |
| mother_unemployed, n (%) | < 5 (< 3.4) | | < 5 (< 9.8) | |
|  | < 5 | < 5 | < 5 | < 5 |
| father_unemployed, n (%) | 5 (3.4) | | < 5 (< 9.8) | |
|  | < 5 | < 5 | < 5 | < 5 |
| mother_mental_disorder (yes), n (%) | 20 (13.7) | | 9 (17.6) | |
|  | < 5 | 16 (19.3) | < 5 | 6 (20.7) |
| father_mental_disorder (yes), n (%) | 5 (3.4) | | < 5 (< 9.8) | |
|  | < 5 | < 5 | < 5 | < 5 |

**Sample preparation for metabolomics analysis**

Plasma samples were processed before liquid chromatography-mass spectrometry (LC-MS) analyses at the Biocenter Kuopio LC-MS metabolomics facility (University of Eastern Finland, Finland). Samples were thawed on ice for 3 h, and 100 μl of each sample was mixed with a pipette with 400 μl of acetonitrile on a 96-well filter plate (Captiva ND Plate 0.2 μm PP, Agilent Technologies, USA) to thoroughly precipitate proteins from the sample. Samples were then centrifuged (700 × g for 5 min, 4°C) and the supernatants were collected to a 96-well plate (96 DeepWell PP Plate, Thermo Fisher Scientific Nunc, Rochester, NY, USA) which was covered (96 Well Cap Natural, Thermo Fisher Scientific Nunc A/S, Roskilde, Denmark). A small portion (2 µl) was pipetted from half of the randomly selected protein-precipitated samples, which were then pooled together to create a quality control (QC) sample. Study samples were randomised before LC-MS analysis.

**Data pre-processing**

Raw spectral data of each mode (HILIC/RP, ESI+/-) were separately pre-processed in Compound Discoverer software (v. 3.3, Thermo Scientific, CA, USA) using the template of Untargeted Metabolomics with Statistics Detect Unknowns with ID using Online Databases and mzLogic. The modified workflow included retention time (RT) alignment, detection of unknown peaks and ion association, peak quality control and filtering, and gap-filling for missing peaks. In addition, it performed QC correction of peak areas for the time-dependent batch effect of detected molecular features, detection of background components, and prediction of elemental composition. (Souza and Patti, 2021).

For spectrum properties, we used a lower RT limit of 0.3 and an upper RT limit of 16. The threshold of the minimum peak intensity for peak alignment was 500,000, as suggested in the protocol (Souza and Patti, 2021). However, the number of aligned peaks in HILIC data was smaller than expected, and both HILIC datasets were re-analysed using a threshold of 250,000 to yield more features. The latter data was included in further processing. To automatically exclude most low-quality peaks, we adjusted the parameters of the peak quality filtering using the Peak Rating filters (threshold 4, number of files 10) and consolidated the signals with a tolerance of mass and retention time of 5 ppm and 0.2 minutes, respectively. Additionally, features were included only if they met two QC criteria: 1) detected in at least 70% of QC samples, and 2) the relative standard deviation (RSD) of a corrected compound area within the QC samples was less than 20%. Otherwise, default settings were used. In addition to the automated workflow, a quality control check for all detected features (chromatographic peaks) was performed manually. Data pre-processing for the semi-targeted approach was performed similarly, but an additional node to accomplish a search against the list of known metabolites, including metabolite name, formula and mass, was added to the template of untargeted metabolomics. Semi-targeted analysis was done only for HILIC and RP positive data using the minimum peak intensity threshold of 250,000. Pre-processed data consisting of QC-corrected peak areas was exported as .csv files for further processing and analysis.

Data from untargeted and semi-targeted mass list analyses were further processed in R (v.4.3.1). Principal component analysis was performed to assess the general quality of data (Figure A.1). To detect possible outliers, we calculated the mean intensity of each sample and visualised log2-mean intensities in boxplots for quality control, blanks, low SDQ and raised SDQ-samples. Two samples (in the low SDQ group) in the HILIC positive data had notably low mean intensity levels, corresponding to the intensity level of blank samples. These two samples were also detected in the principal component analysis (Figure A.1) and were excluded from the data.

The pre-processing workflow in Compound Discoverer included a step (“Fill Gaps”) for handling the low-intensity peaks called “gaps”, which means the peak signals above the detection threshold are found in some (“real peaks”) but missed in other samples. Thus, the data do not include any zero or missing values. Instead, the peak signals below the detection limit (min. peak intensity, later: threshold) are searched back in the raw data and filled with intensity values less than the threshold. We performed threshold filtering to control the total number of low-intensity (below-the-threshold) signals in the data. First, we visualised the proportion of samples with below-the-threshold signals to define the appropriate exclusion rate (Figure A.2). Threshold filtering was performed separately for low and raised SDQ samples to select metabolite features that were measurable with sufficient intensity levels across all samples. The 80% rule is a commonly used method in metabolomics to exclude the metabolites with more than 20% of missing values among all samples or a specified class (e.g. group) (Sun and Xia, 2023). However, less strict 50% (for untargeted data) and 60% (for semi-targeted data) acceptance levels for below-the-threshold signals were selected in this study due to the nature of the data (no zero/missing values) and the discovery phase of the study. Using the filtered data, quantile normalisation was performed to remove the unwanted sample-to-sample variation, followed by log2 transformation to adjust the variance of the different features (Sun and Xia, 2023). Data visualisation was performed using the ggplot2 package (v.3.5.1).


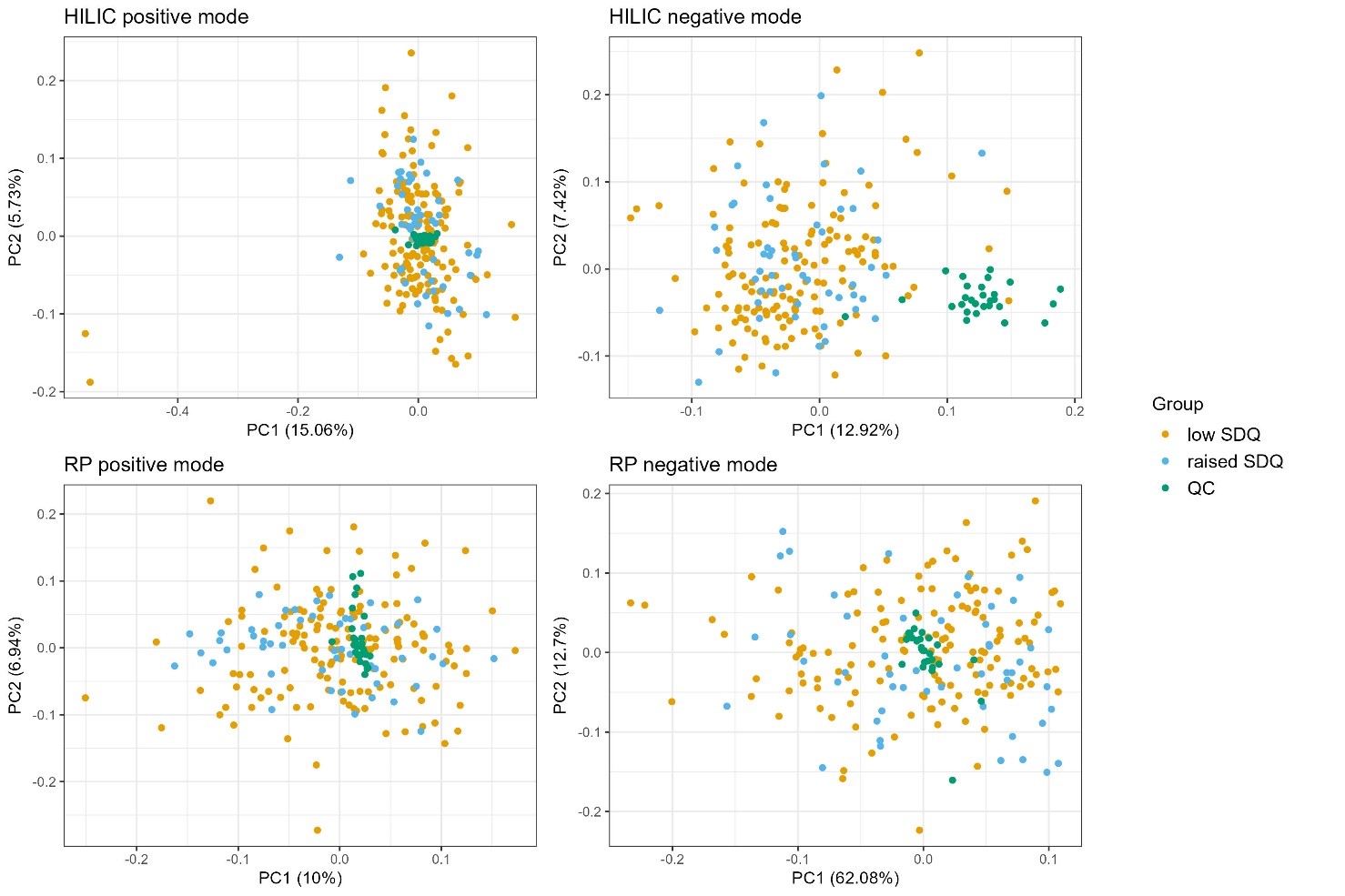


**Figure A.1.** Principal component analysis for the overview of the LC-HRMS data quality. Two outliers were detected and excluded from the analyses in HILIC positive data. Quality control (QC) samples in the HILIC negative data indicated a potential data quality issue in this dataset.

**Statistical analysis**

Data processing and statistical analyses were performed using R (R Core Team, v.4.3.2). The limma (Phipson et al., 2016) package (v.3.56.2) was used for linear regression modelling, with total SDQ scores as a continuous variable and age, sex, BMI, and fasting time as covariates. As a second model to detect possible non-linear associations between the molecular features and the total SDQ score, splines were included in limma modelling using the same covariates. A basis matrix for representing the family of piecewise-cubic splines with 3 nodes was generated using the *ns* function from the total SDQ score (Splines package v. 4.3.1). Moderate *t*- and *F*-test on the SDQ score using the functions *lmfit* and *eBayes* in limma was performed to estimate the significance of linear and non-linear associations of the metabolite intensity with the total SDQ score, respectively. The linear effect size is the log2-fold change in intensity resulting from a unit (1 score) change in the total SDQ score.

**Feature annotations**

In the untargeted LC–MS workflow, all detected features were retained during preprocessing and statistical modelling. Feature grouping and redundancy reduction were performed at the annotation stage. Duplicate or related features were defined as: a) Common adducts (e.g., [M+H]⁺, [M+Na]⁺, [M−H]⁻), b) in-source fragments, c) the same compound detected in both RP and HILIC modes, and d) features sharing highly similar MS/MS spectra, co-elution (within ±X min retention time window) or identical precursor m/z (within ±X ppm). When multiple features corresponded to the same annotated metabolite, a single representative feature was selected for reporting using the following hierarchy: 1) availability and quality of MS/MS spectra, 2) highest annotation confidence (MSI level), 3) strongest spectral match score, 4) best chromatographic peak quality, and 5) biologically appropriate mode preference (e.g., RP for amino acids). All other related features were retained in the raw dataset but excluded from the final reported metabolite table to avoid redundancy.

The key metabolites (pregnenolone sulfate, isoleucine, and LPC 20:1/0:0 were annotated based on: a) accurate mass (within ±X ppm), b) MS/MS spectral matching against public databases including HMDB, MassBank, mzCloud, LipidMaps and PubChem, and c) diagnostic fragment ions consistent with reference spectra. No confirmation with authentic chemical standards was performed.

**Figure A.2.** **Occurrence of the below-the-detection threshold intensity data.** Untargeted data analyses.


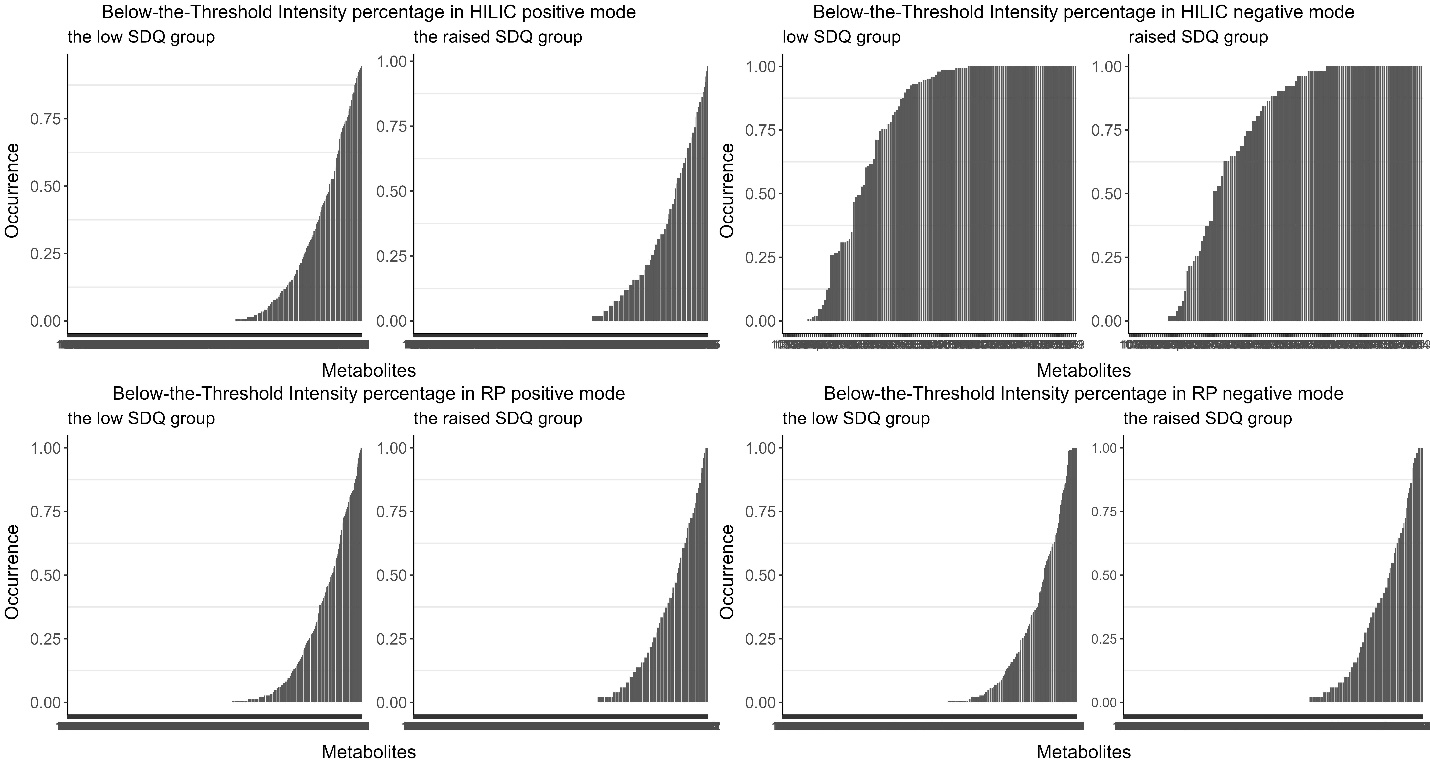


**References**:

Phipson, B., Lee, S., Majewski, I.J., Alexander, W.S., Smyth, G.K., 2016. Robust hyperparameter estimation protects against hypervariable genes and improves power to detect differential expression. Ann. Appl. Stat. 10, 946–963. https://doi.org/10.1214/16-AOAS920.

Souza, A.L., Patti, G.J., 2021. A Protocol for Untargeted Metabolomic Analysis: From Sample Preparation to Data Processing, in: Weissig, V., Edeas, M. (Eds.), Mitochondrial Medicine. Methods in Molecular Biology. Springer, New York, pp. 357–382. https://doi.org/10.1007/978-1-0716-1266-8_27.

Sun, J., Xia, Y., 2023. Pretreating and normalizing metabolomics data for statistical analysis. Genes. Dis. 11, 100979. https://doi.org/10.1016/j.gendis.2023.04.018.
