# Supplementary material for: Plasma metabolomics and red blood cell fatty acid profiles in adolescent mental health
Source: Compr Psychoneuroendocrinol. 2026 Apr 11;26:100347. doi: 10.1016/j.cpnec.2026.100347 (PMC13092191; doi:10.1016/j.cpnec.2026.100347)
Supplement: Multimedia component 3 [file mmc3.docx]

**Supplementary Material C – Results**

**Plasma metabolomics and red blood cell fatty acid profiles in adolescent mental health**

Aino-Kaisa Piironen, Alexey M. Afonin, Iman Zarei, Ville Koistinen, Marko Lehtonen, Venla Hämäläinen, Aleix Sala-Vila, Iolanda Lázaro, Jordi Julvez, Irene van Kamp, and Katja M. Kanninen

**Figure C.1**

**Table C.1**

**Table C.2**

**Table C.3**

**Figure C.2**

**Table C.4**

**Figure C.3**

**Table C.5**

**Table C.6A-C**

**Figure C.4**

**
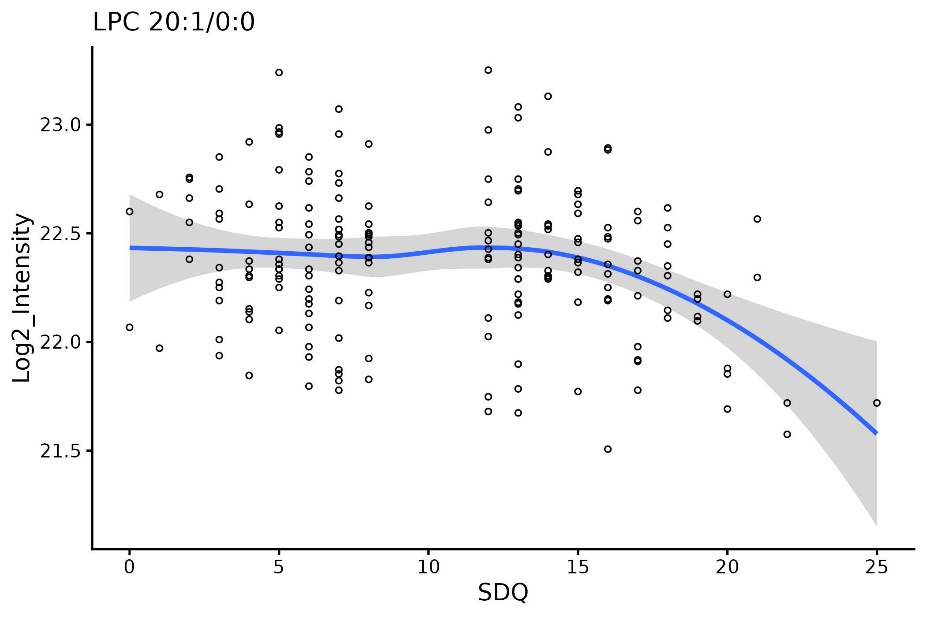

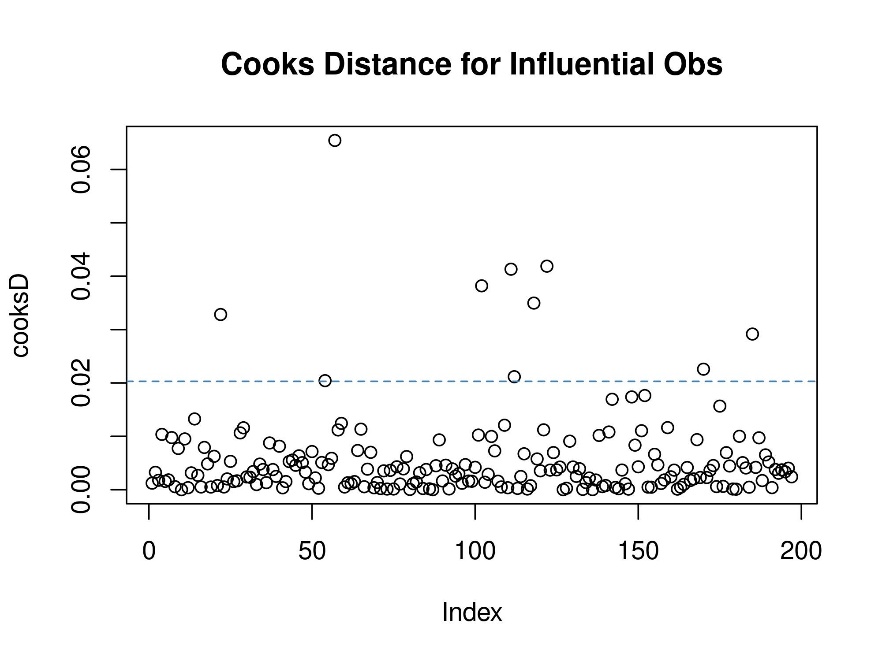
**

**Figure C.1. A non-linear association of long-chain lysophosphatidylcholine LPC 20:1/0:0 with the SDQ score.** The blue line represents the trend line generated by LOESS (Locally Weighted Scatterplot Smoothing), and the grey area indicates the 95% confidence interval. Influential observations in a regression model with a non-linear association, glm(SDQ~ns(LPC 21:0/0:0, df=3)+age+sex+BMI+fasting_time).

**Table C.1.** **Metabolites associated with the total SDQ score in the untargeted analysis (n=197).** Linear effect size is the log2-fold change in intensity resulting from a unit (1 score) change in the total SDQ score.

| **HMDB ID/**  **LIPID MAPS ID** | **Metabolite name** | **MSI ID level** | **ddMS2** | **Mode** | **Calc. MW** | **m/z** | **RT (min)** | **Linear effect size** | **Linear *p*-value** | **Linear *q*-value** | **Non-linear**  ***p*-value** | **Non-linear**  ***q*-value** |
| --- | --- | --- | --- | --- | --- | --- | --- | --- | --- | --- | --- | --- |
| *Metabolites with a linear association with the SDQ score* | | | | | | | | | | | | |
| HMDB0000774,  HMDB0060382/  LMST05020014 | Pregnenolone  sulfate | 2 | PreferredDDA | RP- | 396.198 | 395.190 | 8.812 | -0.038 | 2.8E-5 | 0.029 | 3.5E-4 | 0.079 |
|  | Unnamed *C7H2Cl3OP3S3* | **-** | NoMSn | RP- | 395.755 | 394.747 | 0.504 | 0.011 | 6.0E-5 | 0.041 | 3.8E-4 | 0.079 |
| *Metabolites with a non-linear association with the SDQ score* | | | | | | | | | | | | |
| HMDB0010391 | LPC 20:1/0:0 | 2 | PreferredDDA | RP+ | 549.379 | 550.386 | 10.973 | - | 2.6E-3 | 0.085 | 9.5E-5 | 0.032 |
|  | Unnamed *C50H102N2O14P2 possibly related to LPC 18:1* | - | NoMSn | RP+ | 1016.680 | 1017.687 | 10.571 | - | 6.6E-4 | 0.071 | 3.6E-5 | 0.032 |
|  | Unnamed *C19H36N2O8* | 4 | PreferredDDA | RP- | 420.247 | 419.240 | 11.538 | - | 0.344 | 0.711 | 8.8E-5 | 0.032 |
|  | Unnamed *C31H53N3O8* | 4 | PreferredDDA | RP- | 595.386 | 594.379 | 10.975 | - | 0.011 | 0.176 | 1.2E-4 | 0.035 |

HMDB = the Human Metabolome Database, MSI = Metabolomics Standards Initiative, ddMS2 = data-dependent MS/MS spectra,
SDQ = the Strengths and Difficulties Questionnaire, Calc. MW = Calculated Molecular Weight, m/z = mass-charge ratio, RT = retention time,
LPC = lysophosphatidylcholine, RP+ = Reversed phase chromatography, positive ionisation, RP- = Reversed phase chromatography, negative ionisation.

**Table C.2.** **Metabolite associations with the total SDQ score in semi-targeted mass list analysis (n=197).** Nominally significant associations. Linear effect size is the log2-fold change in intensity resulting from a unit (1 score) change in the total SDQ score.

| **HMDB ID/**  **LIPID MAPS ID** | **Metabolite name** | **MSI ID level** | **ddMS2** | **Mode** | **Calc. MW** | **m/z** | **RT [min]** | **Linear effect size** | **Linear *p*-value** | **Linear *q*-value** | **Non-linear**  ***p*-value** | **Non-linear *q*-value** |  |
| --- | --- | --- | --- | --- | --- | --- | --- | --- | --- | --- | --- | --- | --- |
| Metabolites with a linear association with the SDQ score | | | | | | | | | | | | | |
| HMDB0000172 | **Isoleucine** | 2 | PreferredDDA | RP+ | 131.095 | 132.102 | 1.320 | 0.015 | 2.0E-4 | **0.034** | 1.3E-3 | 0.134 |  |
| HMDB0000687 | Leucine | 1 | PreferredDDA | HILIC+ | 131.095 | 132.102 | 3.987 | 0.009 | 4.4E-3 | 0.188 | 0.019 | 0.428 |  |
| HMDB0000158 | Tyrosine | 1 | PreferredDDA | RP+ | 181.074 | 182.081 | 0.685 | 0.012 | 5.5E-3 | 0.188 | 0.035 | 0.516 |  |
| HMDB0000033 | Carnosine | 2 | OtherDDA | HILIC+ | 226.106 | 227.113 | 5.645 | -0.010 | 7.0E-3 | 0.188 | 0.056 | 0.577 |  |
|  | Unnamed LPC | 4 | PreferredDDA | HILIC+ | 493.316 | 494.323 | 1.159 | -0.013 | 7.5E-3 | 0.188 | 1.5E-3 | 0.134 |  |
| HMDB0000517 | Arginine | 1 | PreferredDDA | RP+ | 174.112 | 175.119 | 0.506 | 0.014 | 0.014 | 0.246 | 0.041 | 0.552 |  |
| HMDB0011128/LMGP01050076 | LPC 0:0/18:0 | 2 | PreferredDDA | HILIC+ | 523.363 | 524.370 | 1.180 | -0.008 | 0.019 | 0.272 | 2.4E-3 | 0.138 |  |
|  | LPC 0:0/16:1 | 2 | PreferredDDA | RP+ | 493.317 | 494.324 | 9.924 | -0.011 | 0.025 | 0.289 | 0.027 | 0.428 |  |
| LMFA07070017 | Carnitine 10:1 | 2 | PreferredDDA | RP+ | 313.225 | 314.232 | 6.903 | -0.017 | 0.037 | 0.375 | 0.125 | 0.624 |  |
| HMDB0002183/ LMFA01030185 | Docosahexaenoic  acid | 1 | PreferredDDA | RP+ | 328.240 | 329.248 | 10.846 | -0.019 | 0.044 | 0.403 | 0.198 | 0.665 |  |
| Metabolites with a non-linear association with the SDQ score | | | | | | | | | | | | | |
| HMDB0000159 | Phenylalanine | 3 | PreferredDDA | RP+ | 165.079 | 166.086 | 1.339 | - | 0.857 | 0.926 | 0.023 | 0.428 |  |
| HMDB0011128/LMGP01050076 | LPC 18:0/0:0 | 2 | PreferredDDA | RP+ | 523.364 | 524.372 | 10.899 | - | 0.915 | 0.959 | 0.046 | 0.552 |  |
| HMDB0061700 | LPC 0:0/18:2 | 2 | PreferredDDA | HILIC+ | 519.332 | 520.339 | 1.114 | - | 0.050 | 0.403 | 0.047 | 0.552 |  |

HMDB = the Human Metabolome Database, MSI = Metabolomics Standards Initiative, ddMS2 = data-dependent MS/MS spectra, SDQ = the Strengths and Difficulties Questionnaire, Calc. MW = Calculated Molecular Weight, m/z = mass-charge ratio, RT = retention time, LPC = lysophosphatidylcholine, RP+ = reversed phase chromatography, positive ionisation, HILIC+ = hydrophilic interaction chromatography, positive ionisation.

**Table C.3. Multiple linear regression of the top candidate metabolites and the SDQ score (n=197).**

|  | **Model 1** | | | | **Model 2** | | | |  |
| --- | --- | --- | --- | --- | --- | --- | --- | --- | --- |
| **Coefficients:** | **Estimate** | **SE** | ***t*-value** | ***p*-value** | **Estimate** | **SE** | ***t*-value** | ***p*-value** | |
| (Intercept) | -5.414 | 45.065 | -0.120 | 0.90 | -0.487 | 44.668 | -0.011 | 0.99 | |
| Isoleucine | 3.450 | 1.204 | 2.866 | **4.6E-3** | 3.465 | 1.200 | 2.888 | **4.3E-3** | |
| Pregnenolone sulfate | -1.958 | 0.530 | -3.694 | **2.9E-4** | -1.768 | 0.520 | -3.399 | **8.2E-4** | |
| LPC 20:1/0:0 | -2.401 | 1.124 | -2.135 | **0.03** | -2.682 | 1.099 | -2.441 | **0.02** | |
| age | 1.537 | 0.395 | 3.890 | **1.4E-4** | 1.525 | 0.392 | 3.888 | **1.4E-4** | |
| sex_F | -0.932 | 0.725 | -1.285 | 0.20 |  |  |  |  | |
| BMI | 0.159 | 0.110 | 1.446 | 0.15 |  |  |  |  | |
| fasting_time | 0.028 | 0.112 | 0.254 | 0.80 |  |  |  |  | |
| *p*-value |  |  |  | **2.7E-7** |  |  |  | **4.2E-8** | |
| Residual SE: 4.985 (189 df), Multiple R^2^: 0.2033, Adj.R^2^: 0.1738, F-statistics: 6.891 (7 and 189 df) | | | | | Residual SE: 4.994 (192 df), Multiple R^2^: 0.1879, Adj.R^2^: 0.171, F-statistics: 11.11 (4 and 192 df) | | | | |

SE = standard error, LPC = lysophosphatidylcholine, BMI = body mass index (kg/m^2^), df = degrees of freedom. Metabolites are expressed as log2 intensities.

**
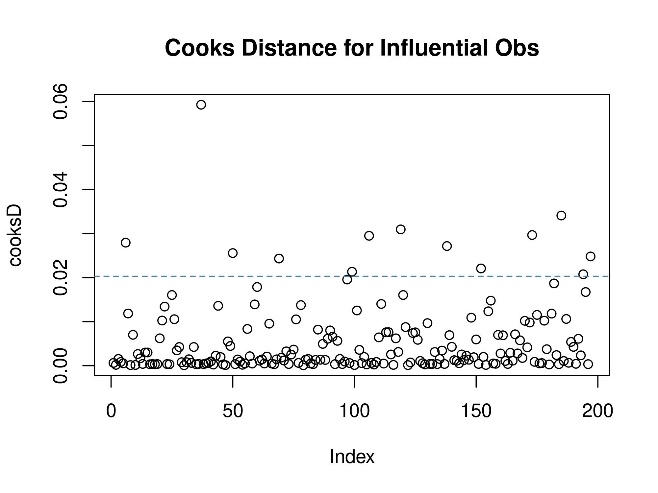

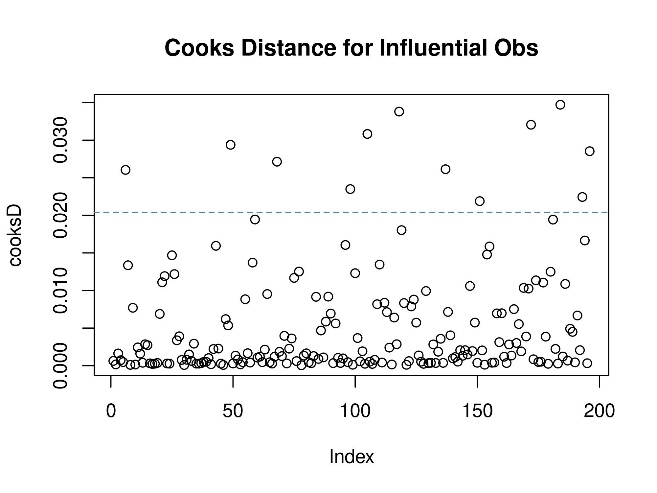

Figure C.2. Cook’s Distance for logistic regression model 5 with all samples (left) and without the potential outlier (right).** Model 5: glm(SDQgroup~isoleucine+pregnenolone sulfate+LPC 20:1/0:0+age, family=binomial)

**Table C.4. Logistic regression model for the top candidate metabolites.**Raised vs low SDQ group, n=196.

| **Model 1** | **Estimate** | **SE** | **z-value** | ***p*-value** | **95 % CI** | **OR** | **AIC** |
| --- | --- | --- | --- | --- | --- | --- | --- |
| (Intercept) | 6.202 | 5.310 | 1.168 | 0.243 |  |  |  |
| Pregnenolone sulfate | -0.582 | 0.241 | -2.414 | 0.016 | [0.35, 0.89] | 0.56 |  |
| age | 0.374 | 0.185 | 2.029 | 0.042 | [1.02, 2.10] | 1.45 | 219.89 |
| **Model 2** |  |  |  |  |  |  |  |
| (Intercept) | -56.271 | 15.742 | -3.575 | 3.51E-4 |  |  |  |
| Isoleucine | 2.021 | 0.610 | 3.313 | 9.22E-4 | [2.37, 26.18] | 7.55 |  |
| age | 0.284 | 0.183 | 1.554 | 0.120 | [0.93, 1.91] | 1.33 | 213.69 |
| **Model 3** |  |  |  |  |  |  |  |
| (Intercept) | 29.735 | 11.679 | 2.546 | 0.011 |  |  |  |
| LPC 20:1/0:0 | -1.610 | 0.530 | -3.039 | 2.37E-3 | [0.07, 0.55] | 0.20 |  |
| age | 0.369 | 0.185 | 1.996 | 0.046 | [1.01, 2.09] | 1.45 | 215.95 |
| **Model 4** |  |  |  |  |  |  |  |
| (Intercept) | -44.410 | 16.778 | -2.647 | 8.12E-3 |  |  |  |
| Isoleucine | 1.953 | 0.620 | 3.151 | 1.63E-3 | [2.17, 24.87] | 7.05 |  |
| Pregnenolone sulfate | -0.524 | 0.246 | -2.130 | 0.033 | [0.36, 0.95] | 0.59 |  |
| age | 0.365 | 0.189 | 1.926 | 0.054 | [1.00, 2.10] | 1.44 | 211.05 |
| **Model 5** | | | | |  |  |  |
| (Intercept) | -8.069 | 22.460 | -0.359 | 0.719 |  |  |  |
| Isoleucine | 1.648 | 0.633 | 2.602 | 9.27E-3 | [1.55, 18.80] | 5.20 |  |
| Pregnenolone sulfate | -0.528 | 0.253 | -2.085 | 0.037 | [0.35, 0.96] | 0.59 |  |
| LPC 20:1/0:0 | -1.317 | 0.564 | -2.337 | 0.019 | [0.08, 0.79] | 0.27 |  |
| age | 0.429 | 0.195 | 2.199 | 0.028 | [1.05, 2.27] | 1.54 | 207.35 |
| **Model 6** |  |  |  |  |  |  |  |
| (Intercept) | -33.143 | 17.338 | -1.912 | 0.056 |  |  |  |
| Isoleucine | 1.589 | 0.636 | 2.499 | 0.012 | [1.46, 17.85] | 4.90 |  |
| Pregnenolone sulfate | -0.574 | 0.258 | -2.223 | 0.026 | [0.34, 0.93] | 0.56 |  |
| ns(LPC 20:1/0:0)1 | -0.588 | 0.792 | -0.743 | 0.458 | [0.12, 2.66] | 0.56 |  |
| ns(LPC 20:1/0:0)2 | -5.214 | 2.483 | -2.100 | 0.036 | [2.9E-5, 0.58] | 0.01 |  |
| ns(LPC 20:1/0:0)3 | -3.575 | 2.005 | -1.784 | 0.075 | [2.3E-4, 0.71] | 0.03 |  |
| age | 0.423 | 0.196 | 2.158 | 0.031 | [1.05, 2.26] | 1.53 | 209.7 |

SE = Standard Error, CI = confidence interval (OR scale), OR = Odds Ratio, AIC = Akaike Information Criterion

**
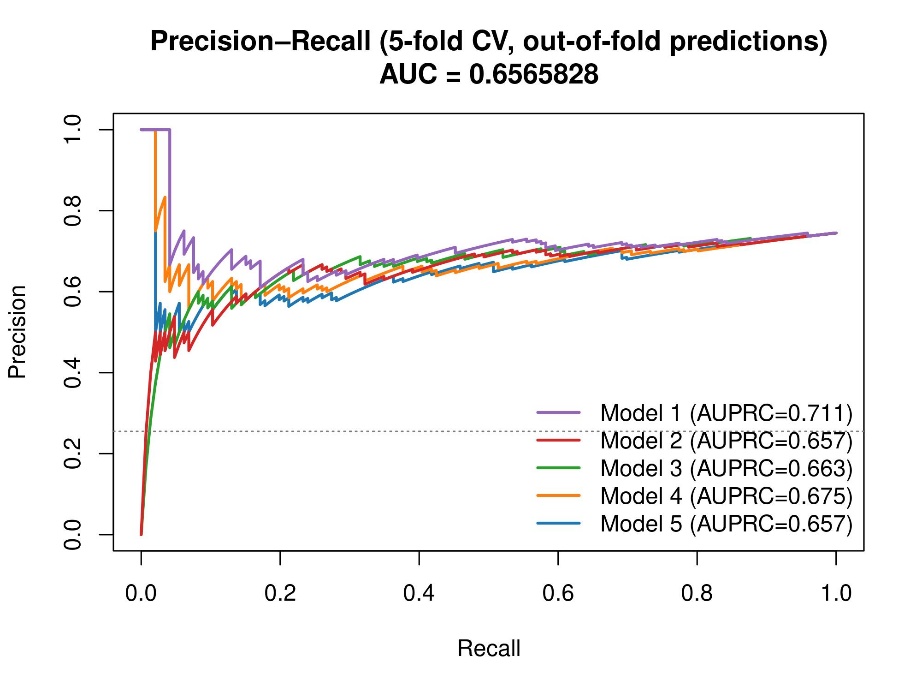
**

**Figure C.3. Precision-Recall curves.** 5-fold stratified cross-validation and out-of-fold prediction for the raised SDQ group using logistic regression models. The horizontal line represents the baseline (0.255), i.e. the prevalence of the raised SDQ group in the data. AUPRC = area under the precision-recall curve.

**Table C.5. Sensitivity analyses for untargeted and semi-targeted mass list metabolomics data (n=197).** Results are presented for candidate metabolites.

| Metabolite | Sensitivity analysis | Linear effect size | Linear  *p*-value | Linear  *q*-value | Non-linear *p*-value | Non-linear *q*-value | *r_s_* | *p*-value |
| --- | --- | --- | --- | --- | --- | --- | --- | --- |
| Isoleucine | Regression, medication | 0.015 | 2.0E-04 | **0.035** | 1.3E-03 | 0.169 |  |  |
|  | Regression, alcohol | 0.016 | 4.4E-04 | **0.029** | 3.7E-03 | 0.217 |  |  |
|  | Regression, smoking | 0.017 | 2.3E-04 | **0.040** | 2.0E-03 | 0.178 |  |  |
|  | Spearman's correlation, fasting time |  |  |  |  |  | -0.043 | 0.545 |
| LPC 20:1/0:0 | Regression, medication | -0.013 | 4.0E-03 | 0.112 | 1.3E-04 | **0.044** |  |  |
|  | Regression, alcohol | -0.012 | 0.012 | 0.277 | 9.0E-04 | 0.180 |  |  |
|  | Regression, smoking | -0.013 | 5.2E-03 | 0.237 | 4.9E-05 | **0.016** |  |  |
|  | Spearman's correlation,  fasting time |  |  |  |  |  | -0.019 | 0.796 |
| Pregnenolone sulfate | Regression, medication | -0.037 | 4.9E-05 | **0.040** | 5.6E-04 | 0.111 |  |  |
|  | Regression, alcohol | -0.037 | 2.2E-04 | 0.155 | 2.2E-03 | 0.232 |  |  |
|  | Regression, smoking | -0.031 | 9.6E-04 | 0.214 | 0.012 | 0.371 |  |  |
|  | Spearman's correlation, |  |  |  |  |  | 0.125 | 0.080 |

**Table C.6A.** Differences in the red blood cell fatty acid compositions between the raised and the low SDQ groups using Welch’s *t*-test and regression analysis. All samples (n=197). Single fatty acids expressed in log2-transformed values. Linear regression models were adjusted for age, sex, and BMI.

|  | **Welch's *t*-test** | | | | | | **Linear regression analysis** | | |
| --- | --- | --- | --- | --- | --- | --- | --- | --- | --- |
| **Variable** | **Estimate** | ***t*-stat** | **df** | **95 % CI** | ***p*-value** | ***q*-value** | **LogFC** | ***p*-value** | ***q*-value** |
| omega-3-index | -0.370 | -2.528 | 89.25 | [-0.66, -0.08] | **0.013** | 0.182 | -0.331 | **0.027** | 0.254 |
| *n-*3 PUFA | -0.383 | -2.264 | 87.28 | [-0.72, -0.05] | **0.026** | 0.182 | -0.338 | **0.047** | 0.270 |
| SFA | 0.163 | 0.837 | 82.69 | [-0.22, 0.55] | 0.405 | 0.954 | 0.137 | 0.474 | 0.932 |
| MUFA | 0.112 | 0.381 | 76.03 | [-0.47, 0.70] | 0.704 | 0.954 | 0.101 | 0.713 | 0.967 |
| *n-*6 PUFA | 0.107 | 0.305 | 79.86 | [-0.59, 0.81] | 0.761 | 0.954 | 0.100 | 0.767 | 0.967 |
| *Single fatty acids* | | | | | | | | | |
| C20_5*n*3 (EPA) | -0.272 | -2.765 | 88.13 | [-0.47, -0.08] | **0.007** | 0.182 | -0.270 | **0.007** | 0.203 |
| C22_6*n*3 (DHA) | -0.115 | -2.310 | 89.57 | [-0.21, -0.02] | **0.023** | 0.182 | -0.101 | **0.048** | 0.270 |
| C24_0 | -0.089 | -2.134 | 117.69 | [-0.17, -0.01] | **0.035** | 0.196 | -0.087 | 0.077 | 0.351 |
| C22_5*n*6 (DPA) | 0.101 | 1.873 | 82.18 | [-0.01, 0.21] | 0.065 | 0.301 | 0.115 | **0.027** | 0.254 |
| C22_4*n*6 | 0.068 | 1.531 | 77.43 | [-0.02, 0.16] | 0.130 | 0.519 | 0.072 | 0.088 | 0.351 |
| C24_1*n*9 | -0.065 | -1.348 | 109.05 | [-0.16, 0.03] | 0.180 | 0.631 | -0.068 | 0.211 | 0.658 |
| C18_1*n*9_trans | -0.024 | -1.177 | 84.62 | [-0.07, 0.02] | 0.242 | 0.754 | -0.030 | 0.151 | 0.529 |
| C14_0 | 0.165 | 1.081 | 73.01 | [-0.14, 0.47] | 0.283 | 0.793 | 0.146 | 0.289 | 0.763 |
| C18_3*n*3 | -0.089 | -0.750 | 85.08 | [-0.32, 0.15] | 0.455 | 0.954 | -0.108 | 0.361 | 0.842 |
| C18_2*n*6 | -0.019 | -0.696 | 83.64 | [-0.07, 0.04] | 0.488 | 0.954 | -0.023 | 0.406 | 0.875 |
| C18_0 | -0.009 | -0.672 | 73.04 | [-0.03, 0.02] | 0.504 | 0.954 | -0.008 | 0.499 | 0.932 |
| C16_1*n*7_trans | -0.089 | -0.613 | 71.70 | [-0.38, 0.20] | 0.542 | 0.954 | -0.133 | 0.300 | 0.763 |
| C18_3*n*6 | -0.050 | -0.581 | 81.03 | [-0.22, 0.12] | 0.563 | 0.954 | -0.040 | 0.627 | 0.967 |
| C18_1*n*9_cis | 0.013 | 0.514 | 76.24 | [-0.04, 0.06] | 0.609 | 0.954 | 0.012 | 0.590 | 0.967 |
| C22_5*n*3 | -0.015 | -0.376 | 73.91 | [-0.10, 0.06] | 0.708 | 0.954 | -0.010 | 0.783 | 0.967 |
| C20_0 | 0.013 | 0.375 | 85.12 | [-0.06, 0.08] | 0.709 | 0.954 | 0.021 | 0.555 | 0.967 |
| C20_4*n*6 | 0.005 | 0.230 | 75.26 | [-0.04, 0.05] | 0.819 | 0.954 | 0.005 | 0.792 | 0.967 |
| C20_1*n*9 | 0.007 | 0.176 | 89.50 | [-0.07, 0.08] | 0.861 | 0.954 | 0.008 | 0.834 | 0.967 |
| C16_1*n*7_cis | -0.008 | -0.104 | 75.49 | [-0.17, 0.15] | 0.917 | 0.954 | -0.014 | 0.842 | 0.967 |
| C22_0 | -0.005 | -0.101 | 78.16 | [-0.11, 0.10] | 0.920 | 0.954 | -0.001 | 0.981 | 0.981 |
| C16_0 | 0.001 | 0.071 | 102.04 | [-0.02, 0.02] | 0.944 | 0.954 | -0.001 | 0.932 | 0.967 |
| C20_2*n*6 | -0.003 | -0.059 | 106.36 | [-0.10, 0.10] | 0.953 | 0.954 | 0.005 | 0.928 | 0.967 |
| C20_3*n*6 | -0.002 | -0.058 | 88.72 | [-0.09, 0.08] | 0.954 | 0.954 | -0.004 | 0.929 | 0.967 |

*n-*3 = omega-3, *n-*6 = omega-6, PUFA = polyunsaturated fatty acids, SFA = saturated fatty acids,
MUFA = monounsaturated fatty acids, EPA = eicosapentaenoic acid, DHA = docosahexaenoic acid, C24_0 = lignoceric acid, DPA= docosapentaenoic acid, *n*-6 isomer.

**Table C.6B.** Differences in the red blood cell fatty acid compositions between the low and the raised SDQ groups in girls (n=85) using Welch’s *t*-test and regression analysis. Single fatty acids expressed in log2-transformed values. Linear regression models included SDQ group x sex-interaction and were adjusted
for age and BMI.

|  | **Welch's *t*-test** | | | | | | **Linear regression analysis** | | |
| --- | --- | --- | --- | --- | --- | --- | --- | --- | --- |
| **Variable** | **Estimate** | ***t*-stat** | **df** | **95 % CI** | ***p*-value** | ***q*-value** | **LogFC** | ***p*-value** | ***q*-value** |
| omega-3-index | -0.652 | -3.086 | 48.49 | [-1.08, -0.23] | **0.003** | 0.069 | -0.595 | **0.009** | 0.154 |
| *n-*3 PUFA | -0.712 | -2.928 | 48.13 | [-1.20, -0.22] | **0.005** | 0.069 | -0.648 | **0.012** | 0.154 |
| MUFA | 0.478 | 0.977 | 27.69 | [-0.52, 1.48] | 0.337 | 0.944 | 0.471 | 0.258 | 0.723 |
| *n-*6 PUFA | 0.245 | 0.471 | 33.59 | [-0.81, 1.30] | 0.640 | 0.994 | 0.232 | 0.652 | 0.909 |
| SFA | -0.012 | -0.032 | 28.30 | [-0.75, 0.72] | 0.975 | 0.999 | -0.056 | 0.848 | 0.938 |
| *Single fatty acids* | | | | | | | | | |
| C22_6*n*3 (DHA) | -0.208 | -2.800 | 46.29 | [-0.36, -0.06] | **0.007** | 0.069 | -0.187 | **0.017** | 0.154 |
| C20_5*n*3 (EPA) | -0.301 | -1.870 | 35.81 | [-0.63, 0.03] | 0.070 | 0.488 | -0.301 | **0.049** | 0.341 |
| C18_0 | -0.034 | -1.609 | 26.86 | [-0.08, 0.01] | 0.119 | 0.565 | -0.034 | 0.061 | 0.341 |
| C22_5*n*3 | -0.100 | -1.594 | 31.22 | [-0.23, 0.03] | 0.121 | 0.565 | -0.094 | 0.083 | 0.388 |
| C24_1*n*9 | -0.119 | -1.429 | 38.97 | [-0.29, 0.05] | 0.161 | 0.644 | -0.124 | 0.134 | 0.535 |
| C24_0 | -0.089 | -1.252 | 39.37 | [-0.23, 0.05] | 0.218 | 0.763 | -0.085 | 0.256 | 0.723 |
| C14_0 | 0.278 | 1.110 | 30.05 | [-0.23, 0.79] | 0.276 | 0.858 | 0.252 | 0.231 | 0.723 |
| C18_1*n*9_cis | 0.036 | 0.903 | 28.78 | [-0.05, 0.12] | 0.374 | 0.952 | 0.036 | 0.300 | 0.763 |
| C20_0 | 0.040 | 0.736 | 31.08 | [-0.07, 0.15] | 0.467 | 0.994 | 0.051 | 0.343 | 0.800 |
| C16_1*n*7_cis | 0.081 | 0.735 | 30.60 | [-0.14, 0.31] | 0.468 | 0.994 | 0.077 | 0.477 | 0.909 |
| C18_3*n*3 | 0.108 | 0.560 | 27.52 | [-0.29, 0.50] | 0.580 | 0.994 | 0.080 | 0.656 | 0.909 |
| C16_1*n*7_trans | 0.128 | 0.529 | 27.10 | [-0.37, 0.62] | 0.601 | 0.994 | 0.063 | 0.746 | 0.909 |
| C22_5*n*6 (DPA) | 0.038 | 0.466 | 33.97 | [-0.13, 0.20] | 0.644 | 0.994 | 0.061 | 0.443 | 0.909 |
| C22_4*n*6 | 0.033 | 0.451 | 29.92 | [-0.12, 0.18] | 0.655 | 0.994 | 0.038 | 0.549 | 0.909 |
| C20_2*n*6 | 0.023 | 0.333 | 34.16 | [-0.12, 0.16] | 0.741 | 0.994 | 0.038 | 0.662 | 0.909 |
| C20_1*n*9 | 0.021 | 0.326 | 27.82 | [-0.11, 0.15] | 0.747 | 0.994 | 0.022 | 0.708 | 0.909 |
| C18_3*n*6 | 0.050 | 0.317 | 25.25 | [-0.27, 0.37] | 0.754 | 0.994 | 0.070 | 0.574 | 0.909 |
| C16_0 | -0.004 | -0.243 | 36.34 | [-0.04, 0.03] | 0.809 | 0.994 | -0.006 | 0.705 | 0.909 |
| C18_2*n*6 | 0.010 | 0.203 | 31.63 | [-0.09, 0.11] | 0.840 | 0.994 | 0.004 | 0.920 | 0.954 |
| C20_3*n*6 | -0.013 | -0.188 | 37.75 | [-0.16, 0.13] | 0.852 | 0.994 | -0.013 | 0.841 | 0.938 |
| C18_1*n*9_trans | -0.002 | -0.075 | 31.44 | [-0.07, 0.06] | 0.941 | 0.999 | -0.011 | 0.732 | 0.909 |
| C22_0 | 0.006 | 0.053 | 29.06 | [-0.21, 0.22] | 0.958 | 0.999 | 0.012 | 0.871 | 0.938 |
| C20_4*n*6 | 0.000 | -0.002 | 35.05 | [-0.07, 0.07] | 0.999 | 0.999 | -9.2E-5 | 0.998 | 0.998 |

*n-*3 = omega-3, *n-*6 = omega-6, PUFA = polyunsaturated fatty acids, SFA = saturated fatty acids,
MUFA = monounsaturated fatty acids, EPA = eicosapentaenoic acid, DHA = docosahexaenoic acid, DPA= docosapentaenoic acid, *n*-6 isomer.

**Table C.6C.** Differences in the red blood cell fatty acid compositions between the low and the raised SDQ groups in boys (n=112) using Welch’s *t*-test and regression analysis. Single fatty acids expressed in log2-transformed values. Linear regression models included SDQ group x sex-interaction and were adjusted for age and BMI.

|  | Welch's *t*-test | | | | | | Linear regression analysis | | |
| --- | --- | --- | --- | --- | --- | --- | --- | --- | --- |
| Variable | Estimate | *t*-stat | df | 95 % CI | *p*-value | *q*-value | LogFC | *p*-value | *q*-value |
| SFA | 0.296 | 1.411 | 66.30 | [-0.12,0.71] | 0.163 | 0.561 | 0.280 | 0.266 | 0.678 |
| omega-3-index | -0.156 | -0.785 | 43.19 | [-0.56, 0.24] | 0.437 | 0.934 | -0.134 | 0.493 | 0.985 |
| *n*-3 PUFA | -0.133 | -0.583 | 42.40 | [-0.59, 0.33] | 0.563 | 0.944 | -0.107 | 0.630 | 0.998 |
| MUFA | -0.165 | -0.451 | 48.82 | [-0.90, 0.57] | 0.654 | 0.944 | -0.175 | 0.627 | 0.998 |
| *n*-6 PUFA | 0.002 | 0.005 | 44.59 | [-0.96,0.96] | 0.996 | 0.996 | 0.001 | 0.998 | 0.998 |
| *Single fatty acids* | | | | | | | | | |
| C22_5*n*6 (DPA) | 0.149 | 2.119 | 48.02 | [0.01,0.29] | **0.039** | 0.561 | 0.156 | **0.023** | 0.518 |
| C20_5*n*3 (EPA) | -0.250 | -2.019 | 50.67 | [-0.50, 0.00] | **0.049** | 0.561 | -0.247 | 0.061 | 0.518 |
| C24_0 | -0.090 | -1.763 | 82.84 | [-0.19, 0.01] | 0.082 | 0.561 | -0.088 | 0.174 | 0.678 |
| C22_4*n*6 | 0.095 | 1.695 | 47.04 | [-0.02,0.21] | 0.097 | 0.561 | 0.097 | 0.081 | 0.518 |
| C18_3*n*3 | -0.238 | -1.603 | 62.05 | [-0.53, 0.06] | 0.114 | 0.561 | -0.248 | 0.111 | 0.518 |
| C18_1*n*9_trans | -0.041 | -1.492 | 52.09 | [-0.10, 0.01] | 0.142 | 0.561 | -0.044 | 0.108 | 0.518 |
| C16_1*n*7_trans | -0.254 | -1.455 | 46.16 | [-0.60, 0.10] | 0.153 | 0.561 | -0.278 | 0.099 | 0.518 |
| C18_3*n*6 | -0.125 | -1.353 | 73.50 | [-0.31, 0.06] | 0.180 | 0.561 | -0.121 | 0.260 | 0.678 |
| C18_2*n*6 | -0.041 | -1.292 | 54.76 | [-0.11, 0.02] | 0.202 | 0.565 | -0.043 | 0.236 | 0.678 |
| C22_5*n*3 | 0.049 | 1.033 | 44.44 | [-0.05,0.15] | 0.307 | 0.781 | 0.053 | 0.259 | 0.678 |
| C18_0 | 0.011 | 0.709 | 49.30 | [-0.02,0.04] | 0.482 | 0.934 | 0.011 | 0.472 | 0.985 |
| C16_1*n*7_cis | -0.076 | -0.688 | 43.90 | [-0.30, 0.15] | 0.495 | 0.934 | -0.082 | 0.381 | 0.889 |
| C22_6*n*3 (DHA) | -0.045 | -0.679 | 44.29 | [-0.18, 0.09] | 0.500 | 0.934 | -0.037 | 0.576 | 0.998 |
| C24_1*n*9 | -0.023 | -0.415 | 75.09 | [-0.13, 0.09] | 0.679 | 0.944 | -0.026 | 0.716 | 0.998 |
| C14_0 | 0.078 | 0.411 | 41.53 | [-0.31,0.46] | 0.683 | 0.944 | 0.068 | 0.709 | 0.998 |
| C16_0 | 0.004 | 0.337 | 66.62 | [-0.02,0.03] | 0.737 | 0.944 | 0.003 | 0.831 | 0.998 |
| C20_2*n*6 | -0.023 | -0.312 | 67.53 | [-0.17, 0.12] | 0.756 | 0.944 | -0.019 | 0.797 | 0.998 |
| C20_4*n*6 | 0.009 | 0.298 | 38.87 | [-0.05,0.07] | 0.767 | 0.944 | 0.009 | 0.726 | 0.998 |
| C22_0 | -0.014 | -0.287 | 61.18 | [-0.11, 0.08] | 0.775 | 0.944 | -0.011 | 0.864 | 0.998 |
| C18_1*n*9_cis | -0.005 | -0.167 | 46.54 | [-0.07, 0.06] | 0.868 | 0.971 | -0.005 | 0.857 | 0.998 |
| C20_0 | -0.006 | -0.134 | 51.71 | [-0.10, 0.09] | 0.894 | 0.971 | -0.002 | 0.971 | 0.998 |
| C20_3*n*6 | 0.006 | 0.107 | 48.89 | [-0.10,0.11] | 0.915 | 0.971 | 0.003 | 0.956 | 0.998 |
| C20_1*n*9 | -0.004 | -0.080 | 67.04 | [-0.10, 0.09] | 0.936 | 0.971 | -0.002 | 0.963 | 0.998 |

*n-*3 = omega-3, *n-*6 = omega-6, PUFA = polyunsaturated fatty acids, SFA = saturated fatty acids,
MUFA = monounsaturated fatty acids, DPA= docosapentaenoic acid, *n*-6 isomer,
EPA = eicosapentaenoic acid, DHA = docosahexaenoic acid.

**
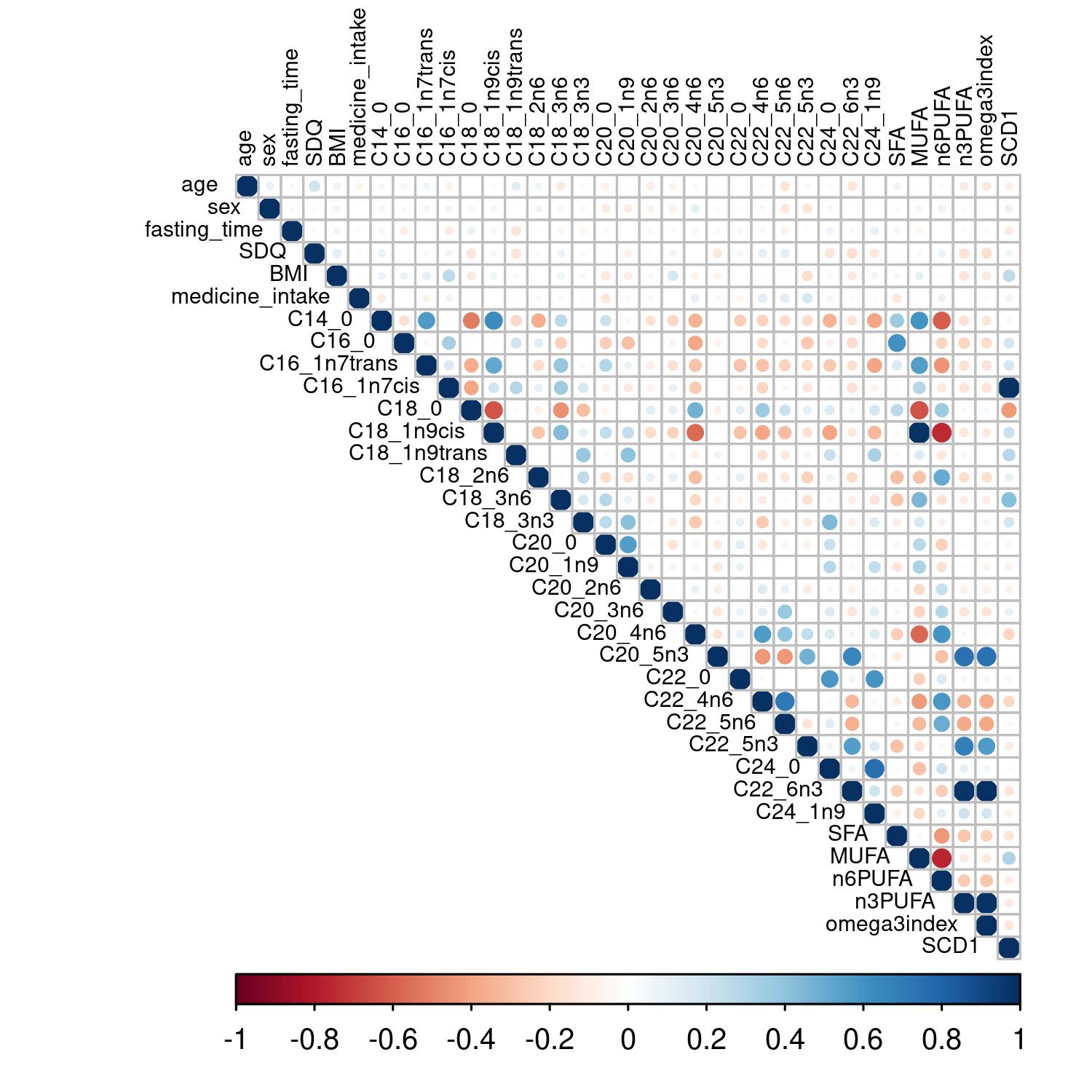
**

**Figure C.4.** **Correlation plot of targeted red blood cell fatty acid measurements and the main confounding factors.** Blue indicates a positive correlation and red a negative correlation.
